# Incremental peritoneal dialysis preserves residual renal function in diabetic end-stage kidney disease

**DOI:** 10.1080/0886022X.2026.2650257

**Published:** 2026-03-31

**Authors:** Lin Lu, Qiaozhi Gu, Yin Zheng, Xiaoye Zhu, Jun Xue, Li You, Dingwei Kuang

**Affiliations:** Department of Nephrology, Huashan Hospital affiliated to Fudan University, Shanghai, China

**Keywords:** Incremental peritoneal dialysis, residual renal function, diabetic kidney disease, end-stage kidney disease, cardiovascular mortality, NT-proBNP

## Abstract

This retrospective cohort study investigated the association between incremental peritoneal dialysis (iPD) and clinical outcomes in 76 diabetic patients with end-stage kidney disease who initiated peritoneal dialysis (PD) between 2013 and 2023. All participants had baseline residual renal function (RRF) ≥3 mL/min/1.73 m^2^. The median age was 67 years, and 71.1% were male. During a median follow-up of 40 months, 44 patients (57.9%) received iPD; 23 (30.3%) lost RRF, 14 (18.4%) transferred to hemodialysis, and 39 (51.3%) died, including 18 (23.7%) from cardiovascular causes. Compared with full-dose PD, iPD was associated with a significantly lower risk of RRF loss in multivariable Cox regression (HR = 0.385, *p* = 0.027) and Fine-Gray competing risk analysis (sHR = 0.384, *p* = 0.029), without increasing the risks of transfer to hemodialysis or mortality. Elevated baseline log-transformed NT-proBNP independently predicted RRF loss (HR = 4.687, *p* = 0.005). Advanced age independently predicted mortality (HR = 1.087 [per years of age], *p* < 0.001), while elevated HbA1c independently predicted both all-cause (HR = 1.641 [per 1% HbA1c], *p* = 0.002) and cardiovascular mortality (sHR = 2.175 [per 1% HbA1c], *p* = 0.002). These findings suggest that iPD is a safe initial strategy that better preserves RRF in diabetic patients with baseline RRF ≥3 mL/min/1.73 m^2^, and optimal glycemic control is crucial for improving long-term outcomes in this high-risk population.

## Introduction

The increasing prevalence of diabetes has led to a growing population of end-stage kidney disease (ESKD) patients. Peritoneal dialysis (PD), an important renal replacement therapy, is witnessing expanded clinical use in many regions, facilitated by policy initiatives and financial incentives [1]. However, long-term survival of PD patients remains suboptimal, with a reported 10-year survival rate of only 11.3% [2]. According to the the European Renal Association – European Dialysis and Transplant Association (ERA-EDTA) registry, the 5-year survival rate for dialysis patients with type 2 diabetes is approximately 30%, significantly lower than the 46% observed in non-diabetic patients [3]. Similarly, data from the 2010 US Renal Data System revealed that while the overall 1- and 2-year survival rates for PD patients were 83% and 67.2%, respectively, these rates dropped to 80.3% and 61.7% in the diabetic subgroup [4]. Thus, improving long-term outcomes remains a substantial challenge for diabetic patients on PD.

To enhance the quality of PD, treatment strategies have been continuously optimized. In 2020, the International Society for Peritoneal Dialysis (ISPD) recommended the use of incremental PD (iPD) as a valid strategy [5]. While a majority of studies suggest that iPD helps preserve residual renal function (RRF) [6–8], the evidence remains inconsistent. For instance, a prospective randomized controlled trial from China reported no significant differences in RRF preservation between patients receiving three versus four daily exchanges of continuous ambulatory peritoneal dialysis (CAPD) [9]. Furthermore, the association between iPD and patient survival remains controversial. While cross-sectional data from Hong Kong showed comparable survival rates between three and four daily 2-liter exchanges of CAPD [10], a multicenter Chinese study reported that patients with ≥ 4 daily exchanges had a significantly lower risk of mortality [11]. Therefore, the impact of iPD on patient prognosis is still a subject of debate.

However, the effect of iPD on clinical outcomes specifically within the high-risk diabetic population has not been fully evaluated. Therefore, this study aims to investigate the impact of iPD on key clinical endpoints, including loss of RRF, transfer to hemodialysis (HD), cardiovascular mortality, and all-cause mortality.

## Materials and methods

### Study design and participants

This retrospective cohort study was conducted in accordance with the ethical principles of the Declaration of Helsinki and approved by the Ethics Committee of Huashan Hospital, Fudan University (Approval No.: KY2016-394). The requirement for informed consent was waived due to the retrospective nature of the study and use of de-identified data.

**Figure 1.**
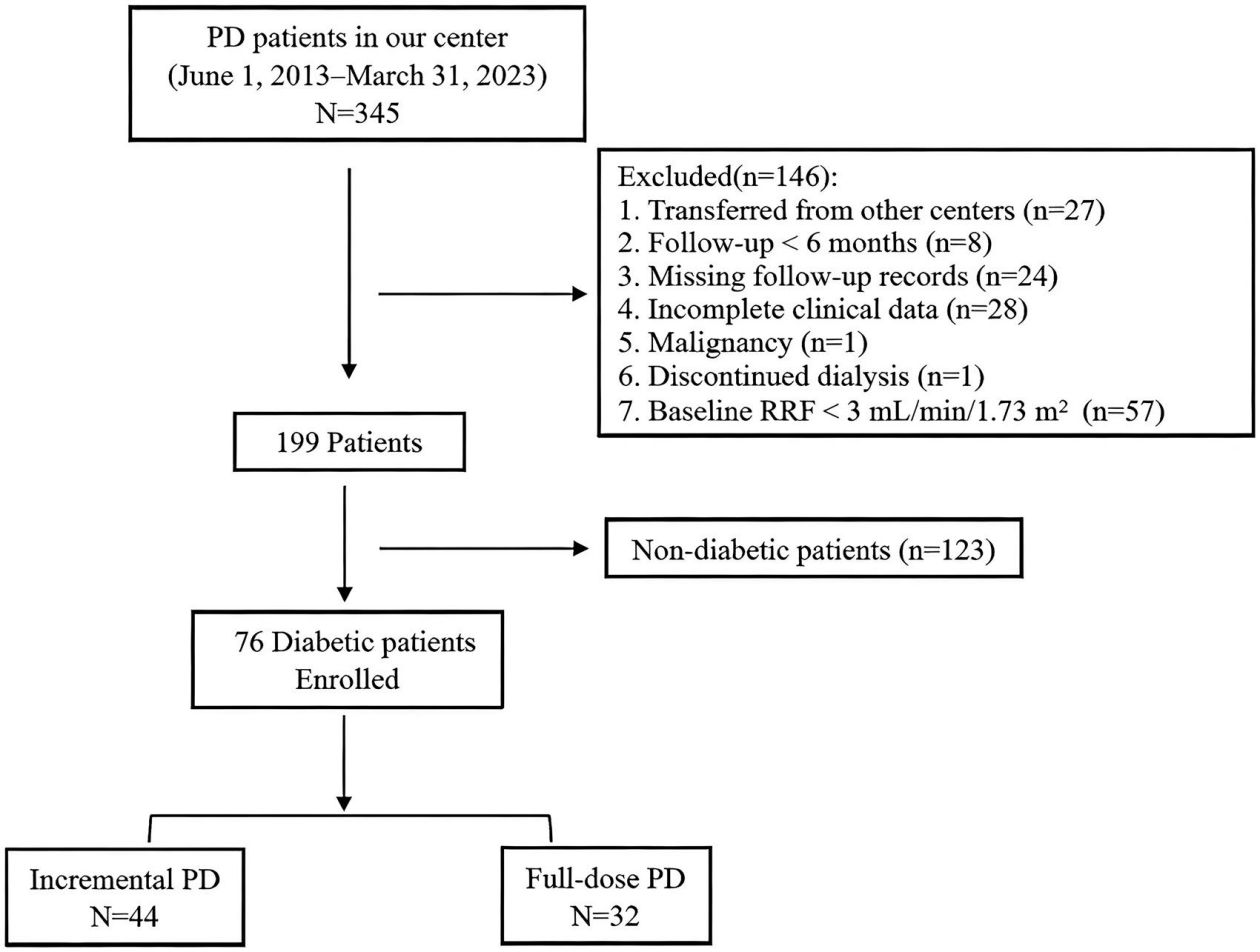
Flowchart of the study population. PD: peritoneal dialysis; RRF: residual renal function.

A total of 76 diabetic patients who initiated PD at the Baoshan Branch of Huashan Hospital were enrolled between June 1, 2013, and March 31, 2023. The inclusion criteria were as follows: (1) adult patients (aged >18 years) undergoing regular PD for at least 6 months; (2) availability of at least two records of PD adequacy assessment; and (3) baseline RRF ≥3 mL/min/1.73 m^2^. Patients were excluded if they met any of the following criteria: (1) incomplete clinical data; (2) receiving PD for acute kidney injury; (3) presence of severe psychiatric disorders, liver diseases, malignancies, or other major comorbidities; (4) non-diabetic status (Figure 1).

### Study groups and dialysis regimens

The exposure status of patients was determined using prescription records from weeks 4 to 6 after PD initiation as the exposure assessment period. This interval was selected to exclude the initial four-week adaptation phase, during which prescriptions were frequently adjusted and may not accurately reflect the long-term treatment regimen.

In this study, patients were categorized into incremental or full-dose PD groups based on baseline RRF, exchange volume, and dialysis modality. Consistent with regional practice in China where three 2-liter exchanges per day (6 L/day) are considered standard full-dose CAPD [10], we used 6 L/day as the dialysis dose threshold to define iPD. To ensure that an incremental approach was clinically appropriate, we restricted the cohort to patients with baseline RRF ≥3 mL/min/1.73 m^2^, a threshold supported by KDOQI guidelines for considering residual kidney function in clearance targets [12]. Within this cohort, we further stratified patients using an RRF threshold of 6 mL/min/1.73 m^2^ to guide regimen selection, based on the Canadian Society of Nephrology guideline suggesting dialysis initiation upon clinical indication or when eGFR drops to ≤6 mL/min/1.73 m^2^ [13]. Accordingly, iPD was defined as:

- Baseline RRF >6 mL/min/1.73 m^2^: Receiving daytime ambulatory peritoneal dialysis (DAPD) with an exchange volume ≤6 L/day, or CAPD with a volume <6 L/day.
- Baseline RRF ≤ 6 mL/min/1.73 m^2^: A total exchange volume ≤6 L/day, irrespective of the dialysis modality (CAPD or DAPD)

**Figure 2.**
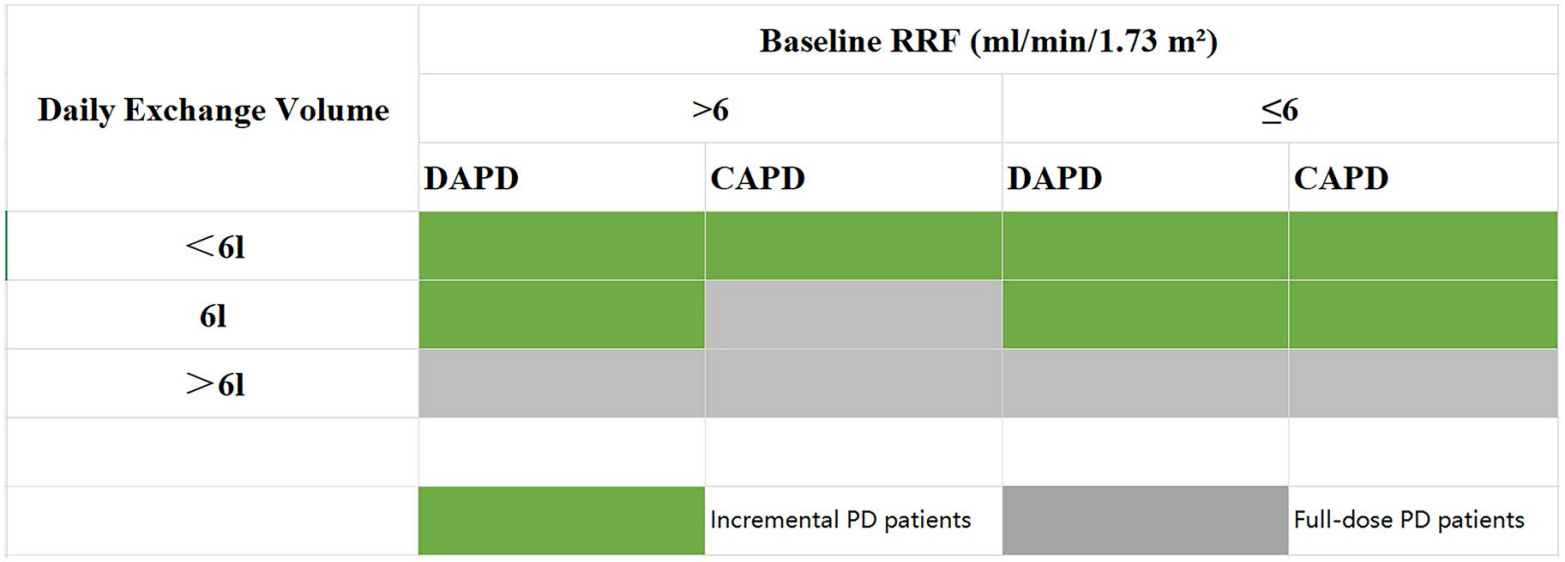
Definition of incremental PD and Full-dose PD regimens according to baseline residual renal function and dialysis prescription. Green shading indicates the incremental PD group; gray shading indicates the full-dose PD group. CAPD: continuous ambulatory peritoneal dialysis; DAPD: daytime ambulatory peritoneal dialysis; PD: peritoneal dialysis; RRF: residual renal function.

Patients who did not meet these specific criteria were assigned to the full-dose group (Figure 2).

This definition aligns with the 2020 ISPD guidelines, which recommend that iPD be tailored to the patient’s RRF^5^.

Our cohort included only patients on manual peritoneal dialysis (CAPD and DAPD); patients on automated peritoneal dialysis (APD) were not enrolled.

During follow-up, regimen adjustments were uniformly managed by two designated physicians based on RRF, clinical presentation, and dialysis adequacy. An increase in the dialysate volume or dwell time was indicated if either of the following criteria was met: (1) weekly total Kt/*V* < 1.70 accompanied by symptoms and/or signs of inadequate dialysis; or (2) fluid overload persisted despite dietary counseling and diuretic therapy.

### Definitions

RRF was calculated as the mean of 24-h urinary urea and creatinine clearances, standardized to a body surface area of 1.73 m^2^. The magnitude of RRF decline during the initial 2-year dialysis period was expressed as the ΔRRF/baseline RRF ratio, with ΔRRF defined as the baseline value minus the RRF at 6, 12, 18, and 24 months. RRF loss was defined as a daily urine output of less than 100 mL. Peritonitis was defined according to the 2022 ISPD guidelines [14], requiring at least two of the following: (1) abdominal pain or cloudy effluent; (2) dialysate WBC >100/μL after ≥2h dwell; (3) positive effluent culture. Transfer to HD was defined as a permanent transition from PD to maintenance hemodialysis (MHD) for a duration of ≥30 days. All-cause mortality was considered an event if it occurred during PD or within 30 days of transfer to HD. The criteria for cardiovascular (CV) death were death attributed to congestive heart failure, acute myocardial infarction, atherosclerotic heart disease, cardiac arrest, cardiac arrhythmia, cardiomyopathy, ischemic or hemorrhagic stroke, anoxic encephalopathy, ischemic brain injury, or peripheral arterial disease.

### Data collection and follow-up

Baseline demographic and clinical data were collected 4–6 weeks after the initiation of PD. These included gender, age, height, weight, primary kidney disease, comorbidities (including diabetes, hypertension, cardiovascular disease [CVD] history), and medication use (including angiotensin-converting enzyme inhibitors [ACEi]/angiotensin II receptor blockers [ARB] and statins). The Charlson Comorbidity Index (CCI) was calculated for all patients.

Laboratory assessments of blood and dialysate samples were performed at baseline and every 6 months. Blood parameters comprised complete blood count, liver and renal function tests, electrolytes, lipid profile, iron indices, parathyroid hormone, N-terminal pro-brain natriuretic peptide (NT-proBNP), fasting glucose, glycated hemoglobin (HbA1c), and C-reactive protein (CRP). Dialysate analysis included biochemical markers and glucose concentration. Additionally, key clinical events and treatment details, including the date of PD catheter insertion, daily urine volume, ultrafiltration volume, and specific PD regimen details, were recorded.

Follow-up was terminated at the time of transfer to HD, kidney transplantation, death, loss to follow-up, or the end of the study (March 31, 2025).

### Statistical analysis

Continuous variables were tested for normality and are presented as mean ± SD (Student’s t-test) or median with interquartile range (Mann-Whitney U test), as appropriate. Categorical variables are expressed as frequencies and percentages and were compared using the chi-square test.

For time-to-event outcomes, different modeling approaches were applied based on the presence of competing risks. The cumulative incidence of RRF loss was estimated using Fine-Gray competing risk models, with death before RRF loss as the competing event; between-group differences were assessed using Gray’s test. For transfer to HD, Fine-Gray models were used with death and kidney transplantation as competing events. For cardiovascular mortality, Fine-Gray models were applied with non-cardiovascular death as the competing event. For all-cause mortality, standard Cox proportional hazards regression was used, as this is the terminal event without competing risks. Results are presented as hazard ratios (HR) with 95% confidence intervals (CI) for Cox models, and subdistribution hazard ratios (sHR) with 95% CI for Fine-Gray models.

Pre-specified multivariable models were constructed incorporating variables with *p* < 0.05 in univariable analysis and clinically relevant factors. For RRF loss, both Cox and Fine-Gray models were adjusted for age, sex, iPD, and log-transformed NT-proBNP. For all-cause mortality, due to potential collinearity between age and CCI, these two variables were not included together in the same model; separate models were built with age or CCI as alternative measures of comorbidity burden. For cardiovascular mortality, three pre-specified Fine-Gray models were constructed with incremental adjustments: Model 1 adjusted for HbA1c and iPD; Model 2 further adjusted for age; Model 3 further adjusted for history of CVD.

To assess the robustness of the iPD effect on RRF preservation, multiple sensitivity analyses were performed with different covariate adjustments. For all-cause mortality, a sensitivity analysis excluding early events (≤12 months) was conducted.

A two-sided P-value < 0.05 was considered statistically significant. All statistical analyses were performed using IBM SPSS Statistics (version 20.0) and R software (version 4.1.0) with the cmprsk package for competing risk analyses.

## Results

### Baseline characteristics of the patients

A total of 76 diabetic patients on PD were included in the study. The median age was 67 (IQR 61–71) years, 71.1% were male and 39.5% had comorbid CVD. Diabetic nephropathy was the primary etiology of kidney failure in 98.7% (75/76) of patients. The median baseline RRF was 6.1 (IQR 4.5–8.4) mL/min/1.73 m^2^. iPD was administered to 44 patients (57.9%). The median follow-up period for the entire cohort was 40 (IQR 28–53) months. Within the iPD group, the median duration of maintaining the incremental regimen was 21 (IQR 14–35) months.

Compared with the full-dose group, the incremental group exhibited a higher proportion of patients treated with DAPD (*p* < 0.001), a lower 4h D/P Cr (*p* = 0.013), and higher serum albumin levels (*p* = 0.022). No statistically significant differences were observed in other baseline characteristics between the two groups (Supplementary Table S1).

### Clinical outcomes

#### Magnitude of residual renal function decline

**Figure 3.**
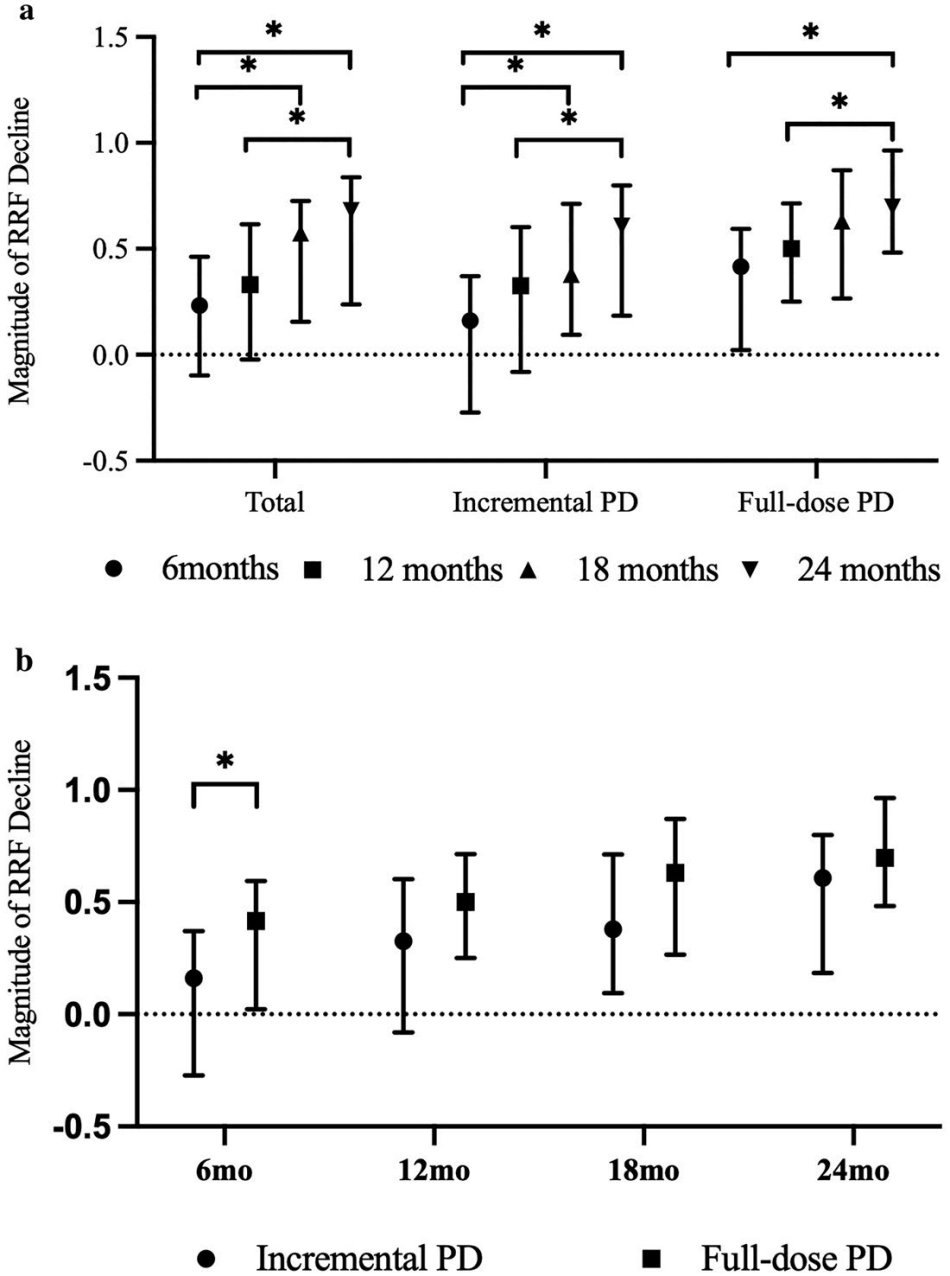
Magnitude of residual renal function decline during the first 24 months of dialysis. (a) Temporal trends of RRF decline within the total cohort, full-dose PD, and incremental PD groups. Statistical significance indicates differences between time points within the same group. (b) Comparison of RRF decline magnitude between the full-dose PD and incremental PD groups at 6, 12, 18, and 24 months. * *p* < 0.05. PD: peritoneal dialysis; RRF: residual renal function.

In this cohort, RRF declined progressively over the first two years of dialysis. In both groups, the magnitude of RRF decline increased with dialysis duration, reaching its minimum at 6 months and peaking at 24 months, with statistically significant differences observed across time points (Figure 3(a)). When comparing the two groups, the magnitude of RRF decline tended to be lower in the iPD group at all time points, although this difference reached statistical significance only at 6 months (*p* < 0.05) (Figure 3(b)). Correspondingly, at 24 months, the iPD group showed better preservation of renal Kt/V and mineral metabolism (lower PTH, higher calcium) compared with the full-dose group (Supplementary Table S2).

#### Loss of residual renal function

A total of 23 patients (30.3%) experienced loss of RRF, including 9 in the incremental group and 14 in the full-dose group. The median time to RRF loss was 31 months (IQR 20–38), with the earliest event occurring at 16 months. In the iPD group, one RRF loss event coincided with dose escalation, and eight occurred after transition to full-dose PD.

**Figure 4.**
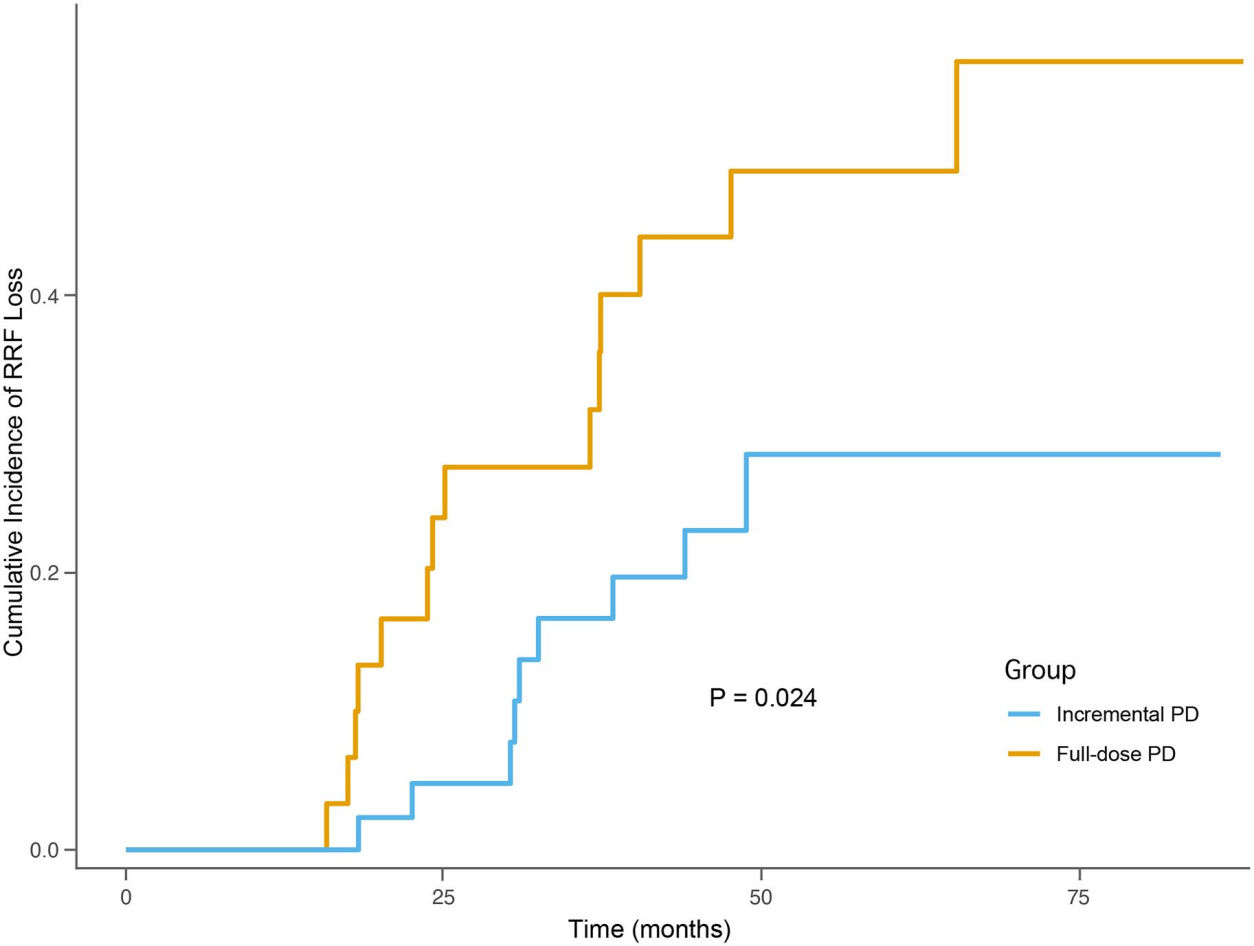
Cumulative incidence of residual renal function loss in patients receiving incremental peritoneal dialysis versus full-dose peritoneal dialysis. Notes: Cumulative incidence curves for RRF loss were estimated using the Fine‑Gray competing risk model with death before RRF loss as the competing event. RRF loss events: iPD, *n* = 9; full‑dose PD, *n* = 14. Competing deaths before RRF loss: iPD, *n* = 16; full‑dose PD, *n* = 8. Abbreviations: PD: peritoneal dialysis; RRF: residual renal function.

In Fine-Gray analysis, patients in the iPD group had a significantly lower cumulative incidence of RRF loss compared with those in the full-dose group (Gray’s test *p* = 0.024; Figure 4). In multivariable analysis adjusting for age, sex, iPD, and log-transformed NT-proBNP, iPD remained independently associated with a lower risk of RRF loss (sHR = 0.384, 95% CI 0.163–0.906, *p* = 0.029). Log-transformed NT-proBNP was also an independent predictor in this model (sHR = 1.940, 95% CI 1.031–3.650, *p* = 0.040) (Table 1).

**Table 1.** Univariable and multivariable Cox regression and Fine-Gray competing risk models for predictors of RRF loss.

Variables	Univariable analysis		Cox model (Model 2)		Fine-Gray model	
	HR (95% CI)	*P* value	HR (95% CI)	*P* value	sHR (95% CI)	*P* value
Incremental PD (vs. full-dose)	0.414 (0.179–0.958)	0.039	0.385 (0.165–0.898)	0.027	0.384 (0.163–0.906)	0.029
log (NT-proBNP)^a^	4.486 (1.543–13.041)	0.006	4.687 (1.607–13.67)	0.005	1.940 (1.031–3.650)	0.040
Male (vs. Female)	1.227 (0.482–3.119)	0.668	1.040 (0.408–2.656)	0.934	1.026 (0.424–2.484)	0.954
Age (per year)	0.986 (0.944–1.029)	0.512	0.994 (0.957–1.033)	0.773	0.976 (0.944–1.009)	0.150
Calcium (per mmol/L)	0.033 (0.002–0.614)	0.024				

Abbreviations: CI: confidence interval; HR: hazard ratio; sHR: subdistribution hazard ratio; RRF: residual renal function; PD: peritoneal dialysis; NT-proBNP: N-terminal pro-B-type natriuretic peptide.

Notes: Both multivariable models were adjusted for sex, age, incremental PD, and log (NT-proBNP).

The Fine-Gray model accounted for death before RRF loss as a competing risk (RRF loss events: *n* = 23; competing deaths before RRF loss: *n* = 24).

log (NT-proBNP) represents Base-10 logarithm of NT-proBNP.

In univariable Cox regression, iPD and higher serum calcium were associated with a lower risk of RRF loss, whereas elevated log-transformed NT-proBNP was associated with an increased risk (all *p* < 0.05). After multivariable adjustment, iPD remained independently associated with a lower risk of RRF loss (HR = 0.385, 95% CI 0.165–0.898, *p* = 0.027), and log-transformed NT-proBNP remained an independent predictor (HR = 4.687, 95% CI 1.607–13.67, *p* = 0.005) (Table 1).

The protective effect of iPD remained consistent across most sensitivity analyses, but was not significant in Model 4b after adjustment for serum calcium (Supplementary Table S3).

#### Transfer to hemodialysis

A total of 14 patients (18.4%) transferred to HD during follow-up (iPD: 8 vs. full-dose: 6), with a median time to transfer of 31 months (IQR 22–48), the earliest transfer occurred at 13 months. The primary causes were inadequate dialysis (*n* = 7) and PD-related peritonitis (*n* = 5), with similar distribution between groups (Supplementary Figure S1a). Peritonitis frequency during follow-up was also comparable between groups (Supplementary Table S4).

In both univariable and multivariable Fine-Gray analyses (Table 2), iPD was not associated with the risk of transfer to HD across all models (all *p* > 0.05). Age was consistently associated with a lower likelihood of transfer (all *p* < 0.05), while baseline RRF showed no significant association.

**Table 2.** Univariable and multivariable Fine-Gray competing risk analyses for transfer to hemodialysis.

	Univariable analysis		Multivariable Model 1		Multivariable Model 2		Multivariable model 3	
Variables	sHR (95% CI)	*P* value	sHR (95% CI)	*P* value	sHR (95% CI)	*P* value	sHR (95% CI)	*P* value
Incremental PD (vs. full‑dose)	1.017 (0.365–2.828)	0.975	1.173 (0.438–3.142)	0.750	0.977 (0.347–2.748)	0.964	1.149 (0.419–3.146)	0.787
Age (per year)	0.952 (0.910–0.997)	0.036	0.951 (0.910–0.995)	0.028			0.953 (0.911–0.997)	0.036
baseline RRF (per mL/min/1.73 m²)	0.960 (0.832–1.107)	0.573			0.959 (0.832–1.107)	0.569	0.968 (0.853–1.098)	0.608

Abbreviations: PD: peritoneal dialysis; RRF: residual renal function; sHR: subdistribution hazard ratio; CI: confidence interval.

Notes: Model 1 includes incremental PD and age. Model 2 includes incremental PD and Baseline RRF. Model 3 includes incremental PD, age, and baseline RRF (exploratory analysis).

All models are Fine‑Gray competing risk models with death (*n* = 38) and kidney transplantation (*n* = 1) as competing events.

#### All‑cause mortality

A total of 39 deaths (51.3%) occurred during follow-up (iPD: 22 vs. full-dose: 17), with a median survival time of 38 months (IQR 25–56). Cardiovascular events were the leading cause of death, followed by infection (Supplementary Figure S1b).

In univariable analysis, older age, higher CCI, and elevated HbA1c levels were associated with an increased risk of all‑cause mortality, whereas higher serum albumin was associated with a reduced risk. Due to strong collinearity between age and CCI (Supplementary Table S5), these two variables were not included together in the same multivariable model. In three pre-specified multivariable Cox models (Table 3), age, CCI, and HbA1c were independent predictors of all-cause mortality, while iPD showed no association in any model. The independent effects of age and HbA1c on all‑cause mortality were robust in a sensitivity analysis excluding the single early death (≤12 months) (Supplementary Table S6).

**Table 3.** Multivariable Cox regression models for predictors of all-cause mortality.

	Univariable analysis		Multivariable model 1		Multivariable model 2		Multivariable model 3	
Variables	HR (95% CI)	*P* value	HR (95% CI)	*P* value	HR (95% CI)	*P* value	HR (95% CI)	*P* value
Incremental PD (vs. full-dose)	1.337 (0.589–2.194)	0.702	1.051 (0.544–2.033)	0.882	1.120 (0.557–2.249)	0.751	1.116 (0.558–2.230)	0.757
HbA1c (per 1%)	1.387 (1.033–1.864)	0.030	1.641 (1.203–2.237)	0.002	1.631 (1.192–2.230)	0.002	1.602 (1.178–2.178)	0.003
Age (per years)	1.069 (1.026–1.114)	0.001	1.087 (1.044–1.132)	<0.001	1.065 (1.021–1.111)	0.003		
CCI (per point)	1.675 (1.215–2.310)	0.002					1.632 (1.184–2.250)	0.003
Male (vs. female)	1.076 (0.778–3.741)	0.182			1.331 (0.567–3.121)	0.511	1.348 (0.563–3.228)	0.502
Albumin (per g/L)	0.907 (0.834–0.986)	0.022			0.946 (0.862–1.039)	0.246	0.930 (0.850–1.017)	0.111
Baseline RRF (per mL/min/1.73 m²)	0.946 (0.878–1.020)	0.149			0.953 (0.873–1.039)	0.275	0.954 (0.875–1.041)	0.290

Abbreviations: CI: confidence interval; HR: hazard ratio; PD: peritoneal dialysis; HbA1c: glycated hemoglobin; CCI: Charlson comorbidity index; RRF: residual renal function.

Note: All models include incremental PD as the primary exposure. Model 1 adjusted for age and HbA1c. Model 2 adjusted for age, HbA1c, sex, baseline RRF, and albumin. Model 3 adjusted for HbA1c, sex, baseline RRF, albumin, and CCI. Due to strong collinearity between age and CCI (Spearman’s ρ = 0.728), these two variables were not included together; Model 2 includes age, while Model 3 includes CCI as an alternative measure of comorbidity burden.

#### Cardiovascular death

A total of 18 cardiovascular deaths (23.7%) occurred during follow-up (iPD: 7 vs. full-dose: 11), with a median time to cardiovascular death of 38 months (IQR 24–62), the earliest event occurred at 17 months.

In univariable Fine-Gray analysis, elevated HbA1c was significantly associated with increased cardiovascular mortality (sHR = 1.646, 95% CI 1.033–2.620, *p* = 0.036), while iPD was not significantly associated with cardiovascular mortality (*p* = 0.075). After multivariable adjustment, HbA1c remained a strong and consistent predictor of cardiovascular mortality across all models (sHR range 2.175–2.208, all *p* < 0.01), whereas iPD, age, and history of CVD were not significantly associated with the outcome (Table 4).

**Table 4.** Univariable and multivariable Fine-Gray competing risk analyses for cardiovascular mortality.

	Univariable analysis		Multivariable model 1		Multivariable model 2		Multivariable model 3	
Variables	sHR (95% CI)	*P* value	sHR (95% CI)	*P* value	sHR (95% CI)	*P* value	sHR (95% CI)	*P* value
Incremental PD (vs. full-dose)	0.440 (0.178–1.087)	0.075	0.409 (0.162–1.035)	0.059	0.400 (0.153–1.045)	0.062	0.404 (0.155–1.053)	0.064
HbA1c (per 1%)	1.646 (1.033–2.620)	0.036	2.175 (1.340–3.531)	0.002	2.187 (1.341–3.566)	0.002	2.208 (1.355–3.598)	0.001
Age (per year)	0.994 (0.941–1.050)	0.822			0.999 (0.938–1.063)	0.967	0.999 (0.938–1.064)	0.983
History of CVD	1.330 (0.543–3.256)	0.212					1.235 (0.457–3.337)	0.678

Abbreviations: sHR: subdistribution hazard ratio; CI: confidence interval; PD: peritoneal dialysis; HbA1c: glycated hemoglobin; CVD: cardiovascular disease.

Note: Model 1 adjusted for HbA1c and Incremental PD; Model 2 adjusted for Model 1 variables plus age; Model 3 adjusted for Model 2 variables plus history of CVD.

All models are Fine‑Gray competing risk models with non‑cardiovascular death as the competing event (cardiovascular death events: *n* = 18; non‑cardiovascular death events: *n* = 21).

## Discussion

In this cohort of diabetic patients initiating PD, iPD was associated with significantly better preservation of RRF compared with full-dose PD, without increasing the risks of all-cause mortality, cardiovascular mortality, or transfer to HD. Baseline NT-proBNP emerged as an independent risk factor for RRF loss, while age and CCI were independent predictors of all-cause mortality, and HbA1c independently predicted both all-cause and cardiovascular mortality.

Compared with non-diabetic counterparts, diabetic patients undergoing dialysis have significantly poorer survival outcomes, with 2.00-fold and 2.11-fold higher risks of all-cause and cardiovascular mortality, respectively [15]. This excess risk is driven by diabetes-specific pathophysiology, including hyperglycemia-induced volume overload and malnutrition [16,17], both of which contribute to earlier dialysis initiation and higher mortality. However, evidence on optimal dialysis strategies for diabetic patients remains limited, highlighting the need for studies specifically targeting this population.

The preservation of RRF is a cornerstone of modern PD management. Sustained RRF facilitates the clearance of uremic toxins, maintains endocrine and volume homeostasis, supports blood pressure control and preserves nutritional status – physiological advantages that translate into improved quality of life and survival [18–24]. Accordingly, RRF is widely recognized as a critical prognostic indicator for PD patients.

In our study, elevated baseline NT‑proBNP was independently associated with RRF loss in multivariable analysis. NT‑proBNP is a well‑established biomarker of ventricular stretch and fluid overload in dialysis patients [25–29]. Its association with RRF loss may reflect the hemodynamic burden and cardiac stress that predispose to renal hypoperfusion and accelerated RRF decline [30]. Of note, the attenuated effect of NT‑proBNP in the competing risk model suggests that part of its prognostic value for RRF loss in standard Cox regression was mediated through the competing risk of death. Additionally, in sensitivity analysis, the protective effect of iPD was attenuated after adjusting for serum calcium, likely due to collinearity between calcium and NT‑proBNP (Supplementary Table S5). Together, these findings highlight NT‑proBNP as a promising biomarker for RRF loss risk in diabetic PD patients, and underscore the importance of considering competing mortality and potential confounding by calcium in such risk assessments.

Consistent with previous research identifying dialysis vintage as a key factor in the decline of RRF [30], our data confirm that RRF loss accelerates during the first two years of therapy. Importantly, patients treated with iPD exhibited a significantly slower rate of RRF decline compared to those on the full-dose regimen. Multivariable analysis confirmed iPD as an independent predictor of reduced RRF loss, an effect that remained robust across multiple sensitivity analyses. These findings extend prior observations in general PD populations [6,31] to the high-risk diabetic setting. Several mechanisms may explain this protective effect in diabetic patients. First, reduced peritoneal glucose exposure may limit advanced glycation end-product (AGE) formation, thereby attenuating chronic inflammation and atherosclerosis [32]. Second, by minimizing glucose absorption from dialysate, iPD may contribute to better glycemic control, which in turn facilitates volume management [33]. Optimal volume status supports residual renal perfusion and preserved RRF further enhances native kidney-mediated fluid excretion [7]. This creating a positive feedback loop that benefits both volume control and renal preservation. Third, chronic high-glucose exposure induces peritoneal fibrosis and ultrafiltration failure. iPD may slow through reduced cumulative glucose load [34]. These interconnected pathways likely act synergistically to slow RRF decline in diabetic patients, with downstream benefits including better preservation of mineral metabolism.

In this study, advanced age and higher CCI were confirmed as independent risk factors for all-cause mortality, consistent with previous reports indicating a higher mortality risk among elderly PD patients [35–38], likely attributable to a greater burden of comorbidities, frailty, and malnutrition [39]. As a composite measure, CCI captures the synergistic effects of multiple coexisting conditions that collectively accelerate mortality risk beyond individual factors, underscoring the importance of comprehensive comorbidity assessment at dialysis initiation to guide personalized treatment strategies.

Interestingly, despite its strong association with mortality, age was inversely associated with transfer to HD in our cohort. This apparent paradox likely reflects the high competing risk of death in older patients: those who might otherwise have required transfer to HD may have died before such transfer could occur. This interpretation is consistent with the Fine-Gray competing risk framework used in our analysis and highlights the necessity of accounting for competing events when evaluating outcomes in elderly dialysis populations.

Elevated HbA1c independently predicted both all-cause and cardiovascular mortality, aligning with previous studies linking poor glycemic control to adverse outcomes in diabetic dialysis patients [40,41]. Mechanistically, uncontrolled hyperglycemia may exacerbate microvascular complications, accelerate peritoneal membrane injury, and promote the progression of advanced diabetic comorbidities. Moreover, as PD is a home-based therapy reliant on self-management, suboptimal glycemic control may serve as a surrogate marker for poor treatment adherence, further compromising prognosis. These findings highlight the need for stringent glycemic management in diabetic patients undergoing PD.

Notably, CVD history was not independently associated with cardiovascular mortality, likely due to limited statistical power due to the modest number of events (*n* = 18) and the high competing risk of non-cardiovascular death (*n* = 21). Furthermore, in diabetic patients with advanced kidney disease, the prognostic impact of traditional CVD risk factors may be overshadowed by diabetes-specific factors such as glycemic control, as evidenced by the strong and consistent association of HbA1c with cardiovascular mortality in our models. Larger studies are warranted to clarify the interplay between CVD history, glycemic control, and cardiovascular outcomes in this population.

This study has several limitations. First, although we applied rigorous multivariable adjustment, competing risk analyses, and multiple sensitivity analyses, the single-center design and modest sample size limit generalizability and statistical power, particularly for outcomes with fewer events. Therefore, these results should be considered hypothesis-generating. Second, despite rigorous adjustment, residual confounding by unmeasured factors cannot be entirely excluded. Data on several clinically relevant parameters, including NSAID and diuretic use, hyperuricemia, proteinuria, erythropoietin dosing, quality of life, left ventricular hypertrophy, and blood pressure management, were unavailable and should be addressed in future studies. Third, the absence of a non-diabetic control group limits the specificity of our conclusions to the diabetic population.

Despite these limitations, this study provides preliminary evidence that iPD reduces RRF loss in diabetic patients (baseline RRF ≥3 mL/min/1.73 m^2^) without increasing mortality or transfer to HD. The robustness of this finding is supported by multiple factors: an extended follow-up (median 40 months; up to 12 years in some patients), a prolonged initial incremental period (median 21 months), and the observation that all RRF loss events in the iPD group occurred only after transitioning to full-dose PD. Given the limited evidence in this high-risk population, our findings contribute to the current understanding of iPD in diabetic patients and provide a basis for future prospective multicenter studies to further evaluate this strategy.

## Conclusion

For diabetic patients initiating PD with baseline RRF ≥3 mL/min/1.73 m^2^, an incremental strategy better preserves RRF without increasing mortality or transfer to HD. These findings suggest iPD may serve as a safe initial strategy in this population. Additionally, optimal glycemic control is crucial for improving long-term outcomes.

## Supplementary Material

Supplemental Material

Supplemental Material

Supplemental Material

## Data Availability

The data underlying this article will be shared on reasonable request to the corresponding author (E-mail: dingweikuang@yeah.net).

## References

[1] Kanjanabuch T, Takkavatakarn K. Global dialysis perspective: Thailand. Kidney360. 2020;1(7):671–675. doi: 10.34067/kid.0000762020. 35372930 PMC8815550

[2] Yangyang X, Xiao Y. Research progress on standardized clinical outcome of peritoneal dialysis. Chin J Nephrol. 2023;39(8):630–634. doi: 10.3760/cma.j.cn441217-20221129-01148.

[3] Stel VS, van de Luijtgaarden MW, Wanner C, et al. The 2008 ERA-EDTA registry annual report-a précis. NDT Plus. 2011;4(1):1–13. doi: 10.1093/ndtplus/sfq191. 21245934 PMC3022422

[4] Collins AJ, Foley RN, Chavers B, et al. United States renal data system 2011 annual data report: atlas of chronic kidney disease & end-stage renal disease in the United States. Am J Kidney Dis. 2012;59(1 Suppl 1):A7, e1-420–A7,420. doi: 10.1053/j.ajkd.2011.11.015. 22177944

[5] Blake PG, Dong J, Davies SJ. Incremental peritoneal dialysis. Perit Dial Int. 2020;40(3):320–326. doi: 10.1177/0896860819895362. 32063212

[6] Sandrini M, Vizzardi V, Valerio F, et al. Incremental peritoneal dialysis: a 10 year single-centre experience. J Nephrol. 2016;29(6):871–879. doi: 10.1007/s40620-016-0344-z. 27582136 PMC5080315

[7] Lee Y, Chung SW, Park S, et al. Incremental peritoneal dialysis may be beneficial for preserving residual renal function compared to full-dose peritoneal dialysis. Sci Rep. 2019;9(1):10105. doi: 10.1038/s41598-019-46654-2. 31300708 PMC6626037

[8] Liu R, Ye H, Peng Y, et al. Incremental peritoneal dialysis and survival outcomes: a propensity-matched cohort study. J Nephrol. 2023;36(7):1907–1919. doi: 10.1007/s40620-023-01735-4. 37603146

[9] Yan H, Fang W, Lin A, et al. Three versus 4 daily exchanges and residual kidney function decline in incident CAPD patients: a randomized controlled trial. Am J Kidney Dis. 2017;69(4):506–513. doi: 10.1053/j.ajkd.2016.08.019. 27751610

[10] Lo WK, Jiang Y, Cheng SW, et al. Survival of CAPD patients in a center using three two-liter exchanges as standard regime. Perit Dial Int. 1996;16(2_suppl):163–166. doi: 10.1177/089686089601601S30. 8728185

[11] Yu X, Chen J, Ni Z, et al. Number of daily peritoneal dialysis exchanges and mortality risk in a Chinese population. Perit Dial Int. 2018;38(Suppl 2):S53–s63. doi: 10.3747/pdi.2017.00283. 30315040

[12] Clinical practice guidelines for hemodialysis adequacy, update 2006. Am J Kidney Dis. 2006;48(Suppl 1):S2–S90. doi: 10.1053/j.ajkd.2006.03.051. 16813990

[13] Nesrallah GE, Mustafa RA, Clark WF, et al. Canadian Society of Nephrology 2014 clinical practice guideline for timing the initiation of chronic dialysis. CMAJ. 2014;186(2):112–117. doi: 10.1503/cmaj.130363. 24492525 PMC3903737

[14] Li PK, Chow KM, Cho Y, et al. ISPD peritonitis guideline recommendations: 2022 update on prevention and treatment. Perit Dial Int. 2022;42(2):110–153. doi: 10.1177/08968608221080586. 35264029

[15] Ma L, Zhao S. Risk factors for mortality in patients undergoing hemodialysis: a systematic review and meta-analysis. Int J Cardiol. 2017;238:151–158. doi: 10.1016/j.ijcard.2017.02.095. 28341375

[16] Van Biesen W, Verger C, Heaf J, et al. Evolution over time of volume status and pd-related practice patterns in an incident peritoneal dialysis cohort. Clin J Am Soc Nephrol. 2019;14(6):882–893. doi: 10.2215/cjn.11590918. 31123180 PMC6556715

[17] Ronco C, Verger C, Crepaldi C, et al. Baseline hydration status in incident peritoneal dialysis patients: the initiative of patient outcomes in dialysis (IPOD-PD study)†. Nephrol Dial Transplant. 2015;30(5):849–858. doi: 10.1093/ndt/gfv013. 25762355 PMC4425480

[18] Diaz-Buxo JA, Lowrie EG, Lew NL, et al. Associates of mortality among peritoneal dialysis patients with special reference to peritoneal transport rates and solute clearance. Am J Kidney Dis. 1999;33(3):523–534. doi: 10.1016/s0272-6386(99)70190-3. 10070917

[19] Paniagua R, Amato D, Vonesh E, et al. Effects of increased peritoneal clearances on mortality rates in peritoneal dialysis: ADEMEX, a prospective, randomized, controlled trial. J Am Soc Nephrol. 2002;13(5):1307–1320. doi: 10.1681/asn.V1351307. 11961019

[20] Termorshuizen F, Korevaar JC, Dekker FW, et al. The relative importance of residual renal function compared with peritoneal clearance for patient survival and quality of life: an analysis of the Netherlands Cooperative Study on the Adequacy of Dialysis (NECOSAD) −2. Am J Kidney Dis. 2003;41(6):1293–1302. doi: 10.1016/s0272-6386(03)00362-7. 12776283

[21] Bargman JM, Thorpe KE, Churchill DN. Relative contribution of residual renal function and peritoneal clearance to adequacy of dialysis: a reanalysis of the CANUSA study. J Am Soc Nephrol. 2001;12(10):2158–2162. doi: 10.1681/asn.V12102158. 11562415

[22] Wang AY, Brimble KS, Brunier G, et al. ISPD cardiovascular and metabolic guidelines in adult peritoneal dialysis patients part i - assessment and management of various cardiovascular risk factors. Perit Dial Int. 2015;35(4):379–387. doi: 10.3747/pdi.2014.00279. 26228782 PMC4520720

[23] Diaz-Buxo JA, White SA, Himmele R. The importance of residual renal function in peritoneal dialysis patients. Adv Perit Dial. 2013;29:19–24. 24344485

[24] Tanriover C, Ucku D, Basile C, et al. On the importance of the interplay of residual renal function with clinical outcomes in end-stage kidney disease. J Nephrol. 2022;35(9):2191–2204. doi: 10.1007/s40620-022-01388-9. 35819749

[25] Goto T, Takase H, Toriyama T, et al. Increased circulating levels of natriuretic peptides predict future cardiac event in patients with chronic hemodialysis. Nephron. 2002;92(3):610–615. doi: 10.1159/000064100. 12372945

[26] Naganuma T, Sugimura K, Wada S, et al. The prognostic role of brain natriuretic peptides in hemodialysis patients. Am J Nephrol. 2002;22(5-6):437–444. doi: 10.1159/000065272. 12381941

[27] Booth J, Pinney J, Davenport A. N-terminal proBNP–marker of cardiac dysfunction, fluid overload, or malnutrition in hemodialysis patients? Clin J Am Soc Nephrol. 2010;5(6):1036–1040. doi: 10.2215/cjn.09001209. 20507952 PMC2879314

[28] Paniagua R, Ventura MD, Avila-Díaz M, et al. NT-proBNP, fluid volume overload and dialysis modality are independent predictors of mortality in ESRD patients. Nephrol Dial Transplant. 2010;25(2):551–557. doi: 10.1093/ndt/gfp395. 19679559

[29] Yamazaki K, Ishii S, Hitaka M, et al. Associations between N-terminal Pro-B-type natriuretic peptide, body fluid imbalance and quality of life in patients undergoing hemodialysis: a cross-sectional study. J Clin Med. 2023;12(23):7356. Nov 28. doi: 10.3390/jcm12237356. 38068408 PMC10706951

[30] Moist LM, Port FK, Orzol SM, et al. Predictors of loss of residual renal function among new dialysis patients. J Am Soc Nephrol. 2000;11(3):556–564. doi: 10.1681/asn.V113556. 10703680

[31] Tu S, Ye H, Xin Y, et al. Early anuria in incident peritoneal dialysis patients: incidence, risk factors, and associated clinical outcomes. Kidney Med. 2024;6(10):100882. doi: 10.1016/j.xkme.2024.100882. 39247762 PMC11380388

[32] Wang AY. Consequences of chronic inflammation in peritoneal dialysis. Semin Nephrol. 2011;31(2):159–171. doi: 10.1016/j.semnephrol.2011.01.005. 21439430

[33] Kim DE, Kim DW, Kim HJ, et al. Impact of glycemic control on residual kidney function and technique failure associated with volume overload in diabetic patients on peritoneal dialysis. Kidney Res Clin Pract. 2025;44(3):481–490. doi: 10.23876/j.krcp.23.251. 39045742 PMC12066335

[34] Del Peso G, Jiménez-Heffernan JA, Bajo MA, et al. Epithelial-to-mesenchymal transition of mesothelial cells is an early event during peritoneal dialysis and is associated with high peritoneal transport. Kidney Int Suppl. 2008;73(108):S26–S33. doi: 10.1038/sj.ki.5002598. 18379544

[35] Couchoud C, Bolignano D, Nistor I, et al. Dialysis modality choice in diabetic patients with end-stage kidney disease: a systematic review of the available evidence. Nephrol Dial Transplant. 2015;30(2):310–320. doi: 10.1093/ndt/gfu293. 25248364

[36] Eldehni MT, Crowley LE, Selby NM. Challenges in management of diabetic patient on dialysis. Kidney Dial. 2022;2(4):553–564. doi: 10.3390/kidneydial2040050.

[37] Hu YH, Liu YL, Meng LF, et al. Selection of dialysis methods for end-stage kidney disease patients with diabetes. World J Diabetes. 2024;15(9):1862–1873. doi: 10.4239/wjd.v15.i9.1862. 39280188 PMC11372645

[38] Klinger M, Madziarska K. Mortality predictor pattern in hemodialysis and peritoneal dialysis in diabetic patients. Adv Clin Exp Med. 2019;28(1):133–135 doi: 10.17219/acem/76751. 30156388

[39] Song YH, Cai GY, Xiao YF, et al. Risk factors for mortality in elderly haemodialysis patients: a systematic review and meta-analysis. BMC Nephrol. 2020;21(1):377. doi: 10.1186/s12882-020-02026-x. 32867718 PMC7457491

[40] Drechsler C, Krane V, Ritz E, et al. Glycemic control and cardiovascular events in diabetic hemodialysis patients. Circulation. 2009;120(24):2421–2428. doi: 10.1161/circulationaha.109.857268. 19948978

[41] Lee MJ, Kwon YE, Park KS, et al. Glycemic control modifies difference in mortality risk between hemodialysis and peritoneal dialysis in incident dialysis patients with diabetes: results from a nationwide prospective cohort in Korea. Medicine (Baltimore). 2016;95(11):e3118. doi: 10.1097/md.0000000000003118. 26986162 PMC4839943

