# Supplementary figures and images for: Incremental peritoneal dialysis preserves residual renal function in diabetic end-stage kidney disease
Source: Ren Fail. 2026 Mar 31;48(1):2650257. doi: 10.1080/0886022X.2026.2650257 (PMC13040572; doi:10.1080/0886022X.2026.2650257)

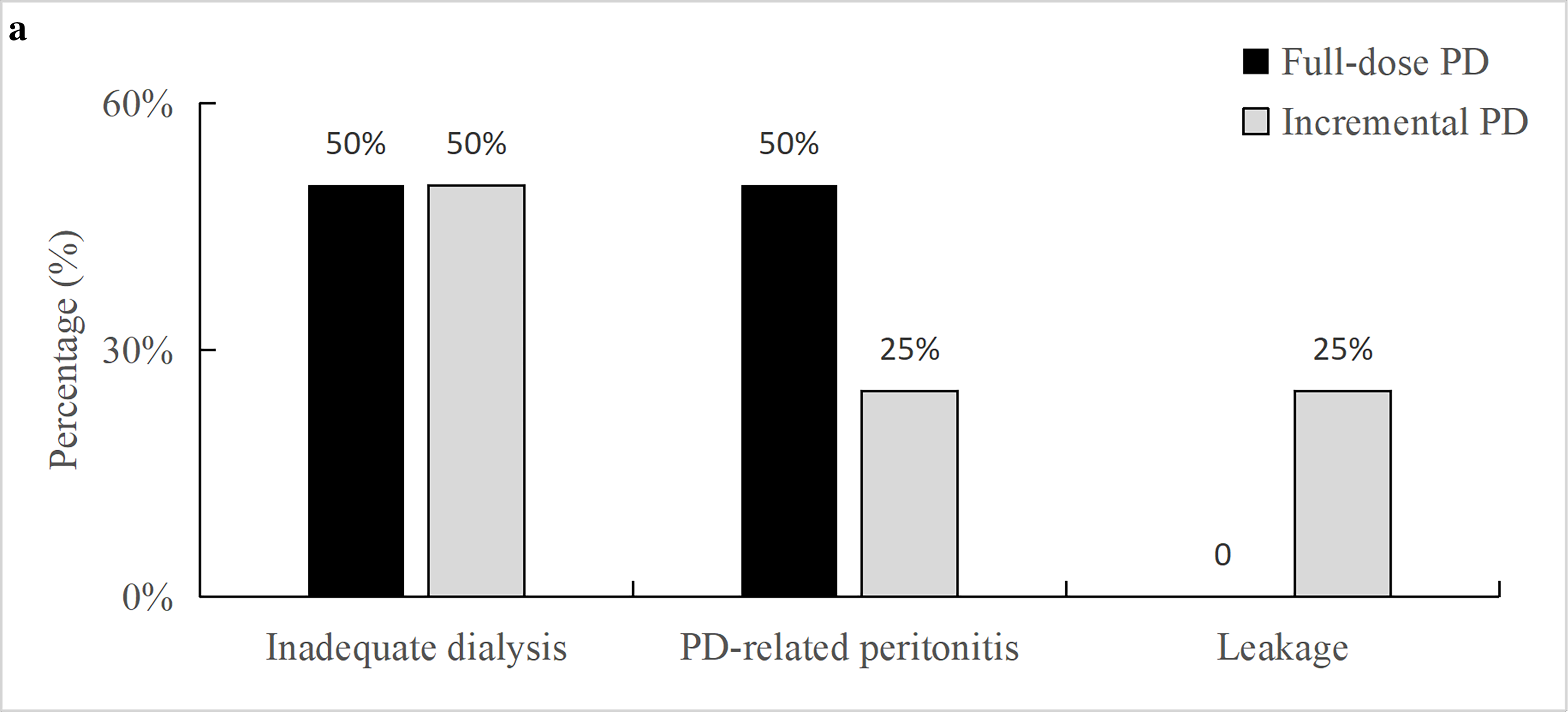

Supplement: Supplemental Material [file IRNF_A_2650257_SM2393.tiff]

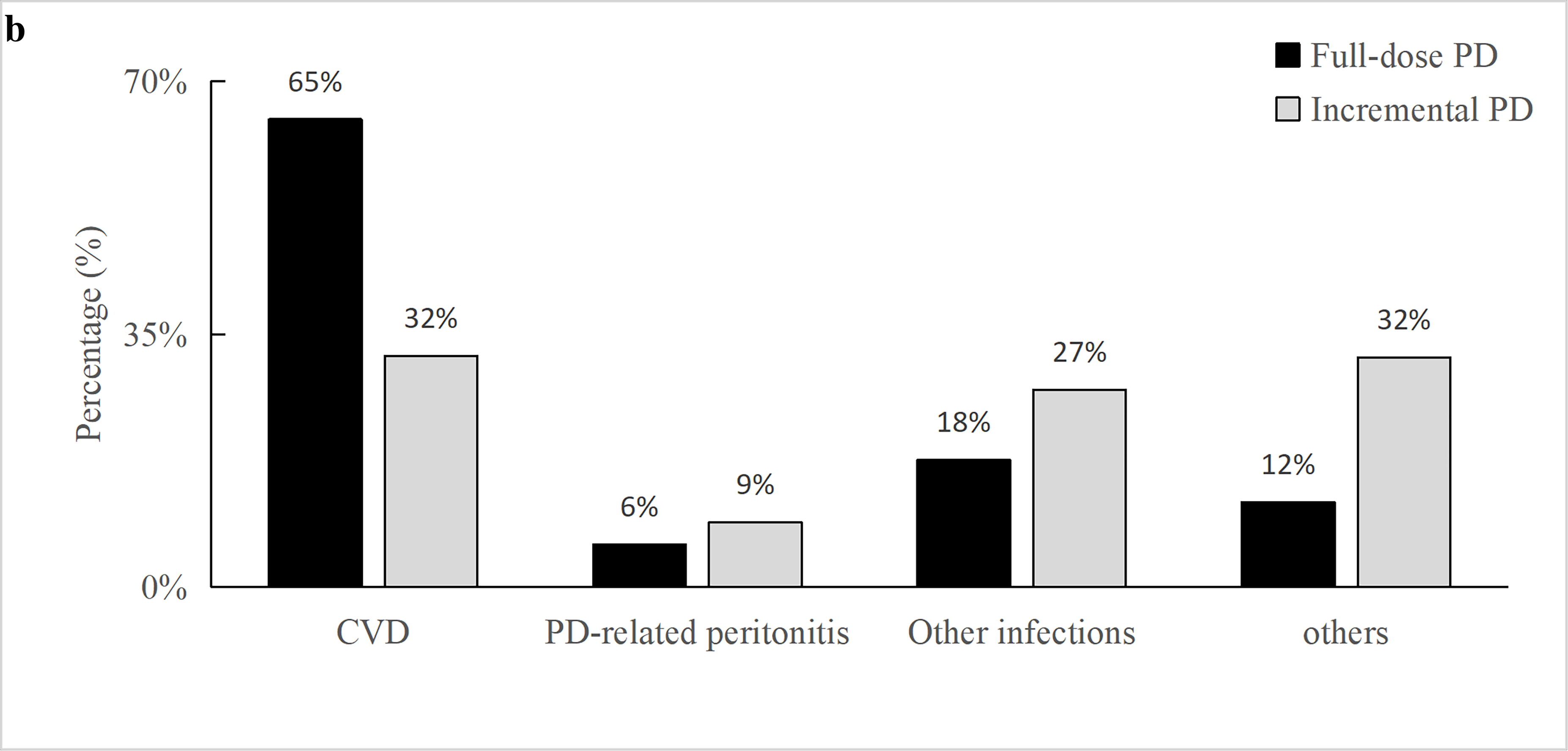

Supplement: Supplemental Material [file IRNF_A_2650257_SM2391.tiff]
