# Supplementary material for: Incremental peritoneal dialysis preserves residual renal function in diabetic end-stage kidney disease
Source: Ren Fail. 2026 Mar 31;48(1):2650257. doi: 10.1080/0886022X.2026.2650257 (PMC13040572; doi:10.1080/0886022X.2026.2650257)
Supplement: Supplemental Material [file IRNF_A_2650257_SM2386.doc]

**T**able S1. Baseline demographic and clinical characteristics of patients stratified by peritoneal dialysis regimen

| **Characteristic** | **Total(n=76)** | **Incremental PD(n=44)** | **Full-dose PD**  **(n=32)** | ***P* value** |
| --- | --- | --- | --- | --- |
| Male, n (%) | 54(71.1) | 30(68.2) | 24(75.0) | 0.518 |
| Age (years) | 67(61,71) | 67(62,71) | 64(57,70) | 0.178 |
| Body Mass Index | 23.92±2.48 | 23.62±2.31 | 24.34±2.67 | 0.224 |
| **History of CVD,** n (%) | 30(39.5) | 17(38.6) | 13(40.6) | 0.861 |
| Charlson Comorbidity Index | 7(6,7) | 7(6,7) | 6(6,7) | 0.115 |
| Medications, n (%) |  |  |  |  |
| ACEi /ARBs | 72(94.7) | 42(95.5) | 30(93.8) | 0.742 |
| Statins | 41(54.0) | 24(54.6) | 17(53.1) | 0.902 |
| Urine volume (mL/d) | 1200(800,1450) | 1150(800,1400) | 1275(750,1500) | 0.446 |
| PD modality, n (%) ^a^ |  |  |  | <0.001 |
| CAPD | 28(36.8) | 8(18.2) | 20(62.5) |  |
| DAPD | 48(63.2) | 36(81.8) | 12(37.5) |  |
| Baseline RRF | 6.1(4.5,8.4) | 6.4(4.9,8.5) | 5.9(4.3,8.4) | 0.871 |
| Baseline Kt/V |  |  |  |  |
| Total KT/V | 2.48(2.13,2.82) | 2.50(2.07,2.80) | 2.47(2.29,2.87) | 0.525 |
| Renal Kt/V | 1.22(0.92,1.64) | 1.24(0.94,1.57) | 1.14(0.85,1.71) | 0.812 |
| Peritoneal Kt/V | 1.23(1.05,1.42) | 1.14(1.02,1.40) | 1.30(1.15,1.43) | 0.068 |
| 4hD/P creatinine ^a^ | 0.67±0.13 | 0.64±0.12 | 0.71±0.12 | 0.013 |
| Hemoglobin(g/L) | 104(95,116) | 104(96,117) | 105(94,113) | 0.626 |
| Albumin(g/L) ^a^ | 31.1(28.1,34.0) | 32.7(28.6,35.3) | 29.9(27.6,32.4) | 0.022 |
| Serum creatinine (μmol/L) | 533(424,637) | 539(422,661) | 522(428,631) | 0.943 |
| Cholesterol(mmol/L) | 3.70(3.01,4.47) | 3.55(2.94,4.41) | 3.76(3.18,4.55) | 0.398 |
| Triglyceride(mmol/L) | 1.52(0.96,2.22) | 1.70(0.96,2.79) | 1.20(0.89,1.88) | 0.179 |
| HDL (mmol/L) | 0.91(0.73,1.11) | 0.87(0.70,1.05) | 1.04(0.81,1.23) | 0.062 |
| LDL (mmol/L) | 1.99(1.50,2.79) | 1.96(1.29,2.69) | 2.45(1.64,2.93) | 0.151 |
| Potassium(mmol/L) | 4.2(3.7,4.7) | 4.3(3.8,4.7) | 4.0(3.6,4.5) | 0.210 |
| Calcium(mmol/L) | 2.16(2.03,2.26) | 2.16(2.08,2.27) | 2.15(1.95,2.23) | 0.278 |
| Phosphorus(mmol/L) | 1.36(1.17,1.62) | 1.36(1.21,1.55) | 1.35(1.16,1.73) | 0.920 |
| iPTH(ng/L) | 151.0(89.9,230.4) | 135.8(71.4,199.3) | 178(104.7,267.2) | 0.135 |
| NT-ProBNP(pg/ml) | 1928(931.2,4690) | 1769(931.2,3254) | 2035(1017.9,5651) | 0.341 |
| HbA1C (%) | 6.5(6.0,7.4) | 6.7(6.3,7.4) | 6.2(5.9,7.2) | 0.180 |
| Creactive protein(mg/L) | 2.80(1.85,5.52) | 2.65(1.75,4.89) | 3.09(1.91,8.68) | 0.396 |

Abbreviations: PD, peritoneal dialysis; CVD, cardiovascular disease; ACEi, angiotensin-converting enzyme inhibitor; ARB, angiotensin II receptor blocker; CAPD, continuous ambulatory peritoneal dialysis; DAPD, daytime ambulatory peritoneal dialysis; RRF, residual renal function; D/P, dialysate-to-plasma; HDL, high-density lipoprotein; LDL, low-density lipoprotein; iPTH, intact parathyroid hormone; NT-proBNP, N-terminal pro-brain natriuretic peptide; HbA1c, glycated hemoglobin.

Data are presented as n (%), median (interquartile range), or mean ± standard deviation.

^a^ indicates a variable with a statistically significant difference (P < 0.05) between the two groups.

Table S2. Clinical parameters at 24 months

| **Parameter** | **Incremental PD** | **Full-dose PD** | **P value** |
| --- | --- | --- | --- |
| Kt/V |  |  |  |
| Total Kt/V | 1.77 (1.52–2.29) | 1.88 (1.55–2.18) | 0.957 |
| Renal Kt/V ^a^ | 0.49 (0.27–0.99) | 0.27 (0.00–0.68) | 0.027 |
| Peritoneal Kt/V ^a^ | 1.20 (1.05–1.46) | 1.37 (1.13–1.68) | 0.048 |
| 4h D/P creatinine | 0.66 ± 0.12 | 0.70 ± 0.11 | 0.192 |
| Hemoglobin (g/L) | 109 (91–113) | 96 (86–103) | 0.077 |
| Albumin (g/L) | 34.2 ± 5.3 | 33.9 ± 4.6 | 0.800 |
| Serum creatinine (μmol/L) | 843 ± 323 | 818 ± 254 | 0.759 |
| Cholesterol (mmol/L) | 3.39 ± 1.12 | 3.60 ± 1.01 | 0.513 |
| Triglyceride (mmol/L) | 1.27 (0.79–2.58) | 1.52 (0.81–3.52) | 0.179 |
| HDL (mmol/L) | 0.87 (0.70–1.05) | 1.04 (0.81–1.23) | 0.062 |
| LDL (mmol/L) | 1.96 (1.29–2.69) | 2.45 (1.64–2.93) | 0.605 |
| Potassium (mmol/L) | 4.1 ± 0.6 | 4.4 ± 1.0 | 0.351 |
| Calcium (mmol/L) ^a^ | 2.17 ± 0.24 | 2.05 ± 0.17 | 0.046 |
| Phosphorus (mmol/L) | 1.66 ± 0.49 | 1.62 ± 0.39 | 0.775 |
| PTH (pg/mL) ^a^ | 199.1 ± 142.0 | 324 ± 156.7 | 0.006 |
| NT-proBNP (pg/mL) | 4332 (992–10275) | 3323 (1393–8724) | 0.891 |
| CRP (mg/L) | 5.82 (2.71–22.4) | 7.30 (1.61–24.3) | 0.971 |
| HbA1c (%) | 7.1 (6.4–7.7) | 7.2 (6.5–7.5) | 0.854 |

Abbreviations: iPD, incremental peritoneal dialysis; D/P, dialysate-to-plasma ratio; HDL, high-density lipoprotein; LDL, low-density lipoprotein; PTH, parathyroid hormone; NT-proBNP, N-terminal pro-brain natriuretic peptide; CRP, C-reactive protein; HbA1c, glycated hemoglobin.

Note: Data are presented as mean ± SD for normally distributed variables or median (interquartile range) for non-normally distributed variables. P values were calculated using Student's t-test or Mann-Whitney U test as appropriate.

^a^ indicates a variable with a statistically significant difference (P < 0.05) between the two groups.

Table S3. Sensitivity analyses for the association between incremental PD and RRF loss

| **Model** | **Adjustment variables** | **HR (95% CI) for iPD** | ***P* value** |
| --- | --- | --- | --- |
| Model 1 | log (NT-proBNP) only | 0.382(0.164–0.889) | 0.026 |
| Model 3 | log (NT-proBNP), Age, Sex, baseline RRF | 0.377(0.161–0.882) | 0.024 |
| Model 4a | log (NT-proBNP), Age, Sex, baseline RRF, Alb | 0.379(0.161–0.893) | 0.026 |
| Model 4b | log (NT-proBNP), Age, Sex, baseline RRF, Ca | 0.488(0.202–1.179) | 0.111 |
| Model 5 | Stepwise selection | 0.426(0.183–0.992) | 0.048 |

Abbreviations: CI, confidence interval; HR, hazard ratio; iPD, incremental peritoneal dialysis; RRF, residual renal function; NT-proBNP, N-terminal pro-B-type natriuretic peptide;Alb, albumin; Ca, calcium.

Notes: All models included incremental PD as the primary exposure. Model numbers correspond to the main analytical framework; Model 2 is presented in Table 1 (multivariable Cox model for RRF loss). log (NT-proBNP) represents base-10 logarithm of NT-proBNP. In Model 4b, additional adjustment for serum calcium attenuated the iPD effect, likely due to collinearity between calcium and NT-proBNP (see Table S5). Model 5 was an exploratory stepwise selection analysis (entry P < 0.05, removal P > 0.10), which retained only incremental PD and log (NT-proBNP) as independent predictors, further supporting the robustness of the primary findings.

Table S4. Peritonitis episodes during follow-up

| **Group** | **Patients with ≥1 peritonitis episode, n (%)** | **Total patients** | ***P* value** |
| --- | --- | --- | --- |
| Incremental PD | 16 (36.4) | 44 |  |
| Full-dose PD | 15 (46.9) | 32 |  |
| Total | 31 (40.8) | 76 | 0.357 |

Abbreviations: PD, peritoneal dialysis.

Note: *P* value was calculated using the chi‑square test for comparison between the incremental PD and full-dose PD groups.

Table S5. Spearman correlation matrix among key continuous variables

| **Variables** | **Age** | **CCI** | **Calcium** | **Albumin** | **log (NT-proBNP)** |
| --- | --- | --- | --- | --- | --- |
| Age | 1.000 |  |  |  |  |
| CCI | 0.728** | 1.000 |  |  |  |
| Calcium | -0.076 | -0.012 | 1.000 |  |  |
| Albumin | -0.200 | -0.172 | 0.547** | 1.000 |  |
| log (NT-proBNP) | 0.039 | 0.211 | -0.325** | -0.469** | 1.000 |

Abbreviations: CCI, Charlson comorbidity index; NT-proBNP, N-terminal pro-B-type natriuretic peptide.

Notes: Spearman correlation coefficients (ρ) are shown. **P < 0.01. log (NT-proBNP) represents base-10 logarithm of NT-proBNP.

**Table S6. Sensitivity analysis for all-cause mortality excluding early events (≤12 months)**

| **Variables** | **Multivariable Model** | |
| --- | --- | --- |
|  | **HR (95% CI)** | ***P* value** |
| Incremental PD (vs. full‑dose) | 1.003(0.515–1.953) | 0.993 |
| Age (per year) | 1.091(1.047–1.136) | <0.001 |
| HbA1c (per 1%) | 1.656(1.207–2.273) | 0.002 |

Abbreviations: HR, hazard ratio; CI, confidence interval; PD, peritoneal dialysis; HbA1c, glycated hemoglobin.

Note: Multivariable Cox regression model excluding one death occurring within 12 months (at 7 months). Model adjusted for age, HbA1c, and incremental PD.
